# A new species of *Physoctonus* Mello-Leitão, 1934 from the ‘Campos formations’ of southern Amazonia (Scorpiones, Buthidae)

**DOI:** 10.3897/zookeys.711.20187

**Published:** 2017-10-23

**Authors:** Wilson R. Lourenço

**Affiliations:** 1 Muséum national d’Histoire naturelle, Sorbonne Universités, Institut de Systématique, Evolution, Biodiversité (ISYEB), UMR7205-CNRS, MNHN, UPMC, EPHE, CP 53, 57 rue Cuvier, 75005 Paris, France

**Keywords:** Campos of Amazonia, disrupt distribution, new species, *Physoctonus*, scorpion

## Abstract

Further studies on new specimens of the rare genus *Physoctonus* Mello-Leitão, 1934, lead to the description of a third new species. Until now only *Physoctonus debilis* (C. L. Koch, 1840) and *Physoctonus striatus* Esposito et al., 2017, were known from sites located in the caatingas of the north-east region of Brazil. The new species of *Physoctonus* was collected by the French arachnologist J. Vellard in the Campos do Pará during his field trips back to the 1920/1930, and entrusted to the author in the early 1980s. The populations of *P. debilis* and *P. striatus* from north-east Brazil and that of the new species certainly present disrupted distributions. Biogeographical comments on this pattern of distribution are also added.

## Introduction

**Figure 1.**
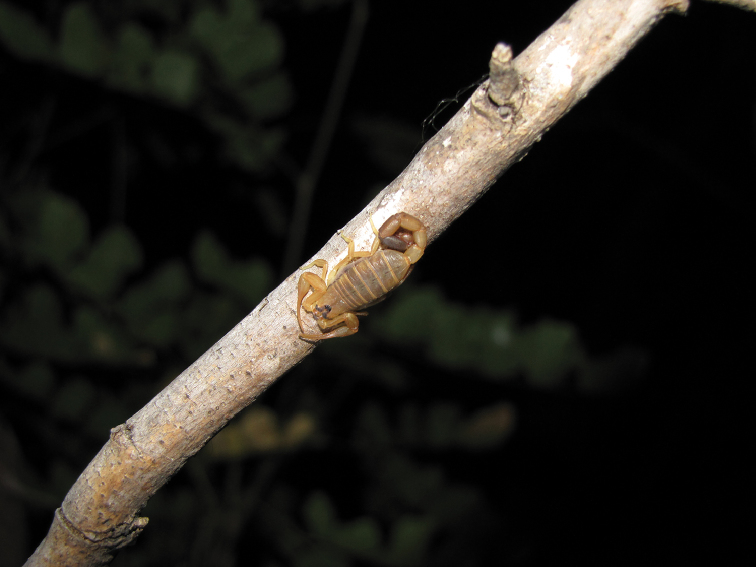
*Physoctonus debilis*, female from Juazeiro, Bahia, Brazil, in natural habitat (photograph Tiago J. Porto).

The two known species belonging to the genus *Physoctonus*, *P. debilis* (C. L. Koch, 1840) and *P. striatus* Esposito et al., 2017 are found exclusively in sites of the north-east region of Brazil (Fig. 1), but remain uncommon (Lourenço 2007; Porto et al. 2014; Esposito et al. 2017). For several decades, *Physoctonus debilis* was associated to the genus *Rhopalurus* Thorell, 1876 as *Rhopalurus debilis*, but for long was also the subject of great taxonomic confusion. The species was originally described in the genus *Vaejovis*, and later included in the family Buthidae by Kraepelin (1899) (as *incertae sedis*). Subsequently, it was transferred to the genus *Rhopalurus* by Borelli (1910) who was able to examine a single specimen. A few decades later, Mello-Leitão (1934) proposed a new genus *Physoctonus* to accommodate a new species *Physoctonus physurus* Mello-Leitão, 1934 from Brazil. In this study Mello-Leitão (1934) apparently ignored *Vaejovis debilis*. In a note concerning several amendments, Francke (1977) discussed the status of *Physoctonus physurus*. He clearly demonstrated that this species was a junior synonym of *Rhopalurus debilis*, and attributed the taxonomic errors cited above to the scarcity of the known material belonging to this species. The decisions taken by Francke (1977) appeared to be justified and were not re-discussed for quite many years. SEM (Scanning Electron Microscopy) studies in the 1990s started to be available for several species of the genus *Rhopalurus* (Lourenço and Cloudsley-Thompson 1995; Lourenço et al. 2000) bringing further evidence about the importance of the stridulatory apparatus in the taxonomy of this genus. However, due to the scarcity of the available material, not all species of *Rhopalurus* were examined with the use of SEM or light microscopy. A few years ago, specimens of *R. debilis* became available for studies with the use of SEM microscopy (Lourenço 2007). The examination of the pectines of this species leads to surprising results since it was found that *R. debilis* lacked any stridulatory apparatus, therefore excluding it from the genus *Rhopalurus*. Moreover, the distribution of the rows of granulations on the pedipalpal chela fingers showed a quite distinct pattern from those observed for the species of *Rhopalurus*. These results lead to the revalidation of the genus *Physoctonus* Mello-Leitão, 1934, described for the single species *P. physurus* (a junior synonym of *R. debilis*). The species described by Koch was consequently placed in a new combination, *Physoctonus debilis* (C. L. Koch, 1840). Since the publication of SEM studies (Lourenço 2007) the genus *Physoctonus* remained monotypic. In an unpublished thesis Yamaguti (2011) suggested the existence of a new species from a region of Caatinga formation in the city of Oeiras in the north-east state of Piauí. Finally, this presumably new species was recently described by Esposito et al. (2017). In this contribution, one new species of *Physoctonus*, collected in the Amazonian Campos of Pará is described.

## The field trips performed by J. Vellard in Brazil

As outlined in previous publications (Lourenço 2001a, 2016), among the zoologists who worked in South America during the first half of the 20^th^ century, the name of Jean A. Vellard is often poorly known or even totally neglected by arachnologists. In fact, his contribution to the study of scorpions was limited to two publications (Vellard 1932, 1934); however, the results he obtained remain very accurate and have been often confirmed in subsequent publications (see Lourenço 2001a, 2016).

**Figure 2.**
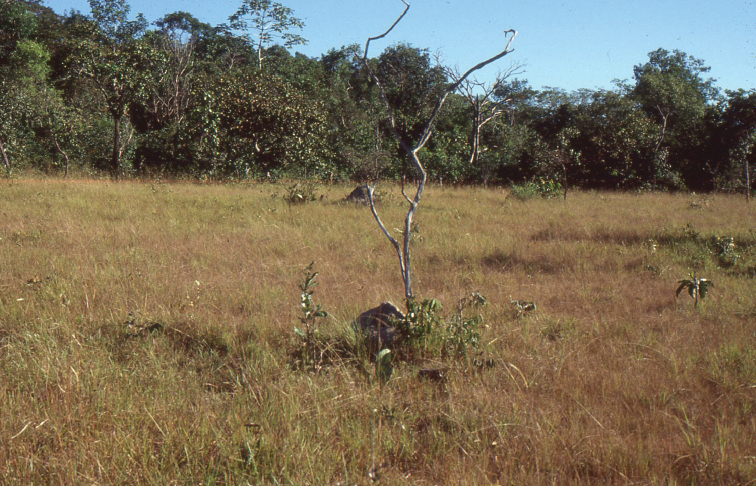
Campos of Pará in southern Amazonia, natural habitat of the new species. Photograph taken by the author in 1979. The region was largely threatened in recent years by anthropic action.

Among the field trips that J. Vellard made in Brazil, one was quite long and important. By the end of the 1920s he left the town of Goyaz (Goiás Velho) in the state of Goiás and navigated to the north along the rivers Araguaia and Tocantins. All this travel was done inside totally unexplored regions at the time. One may recall that the Araguaia River is a tributary of the Tocantins River which is itself a tributary of the Amazon River. During this long trip J. Vellard was able to visit and collect in regions such as the north of Goiás (now state of Tocantins), the south of Pará, in the Campos of Pará (Fig. 2), also known as ‘Campos dos Cayapos’, north of the state of Mato Grosso and particularly in the biggest fluvial island of the world (Bananal) where he dedicated a long period studying venomous fishes (genus *Taeniura*, now *Potamotrygon*) and scorpions (Lourenço 2001a; Adler 2007). J. Vellard described only four new species of scorpions (Vellard 1932, 1934), but all remain valid today. By the end of the 1970s and again in the 1980s field trips were conducted over the same regions previously visited by J. Vellard (Lourenço 2001a) and three of the four species described by him were collected. Nevertheless during the 50–60 years separating our field trips, the natural habitats of these scorpion species suffered intensely due to anthropic activities, in particular cattle grazing. During the north winter of 1980, I had the opportunity to meet J. Vellard in Paris, and he entrusted me a number of scorpion specimens collected in the 1920–1930 period, but never studied. One of these specimens, a new species of *Tityus* C. L. Koch, 1836, was recently described from the state of Tocantins (Lourenço 2016).

The new *Physoctonus* described here is also one of the scorpions Vellard entrusted to the author almost 40 years ago.

## Materials and methods

Illustrations and measurements were obtained using a Wild M5 stereomicroscope with a drawing tube and ocular micrometre. Measurements follow Stahnke (1970) and are given in millimetres. Trichobothrial notations follow Vachon (1974) and morphological terminology mostly follows Hjelle (1990).

**Figure 3.**
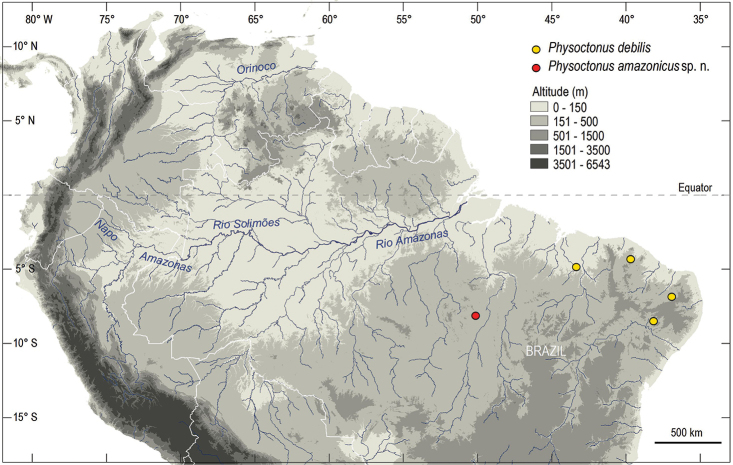
Map of northern South America, showing only the locations in north-east Brazil from where *P. debilis* was studied, and the type locality of the new species.

Several specimens of *Physoctonus debilis* (Koch) have been examined in detail (Fig. 3). The male holotype of *Physoctonus physurus* Mello-Leitão, 1934 (= *Physoctonus debilis*), Santa Luzia, Paraíba state, Brazil, (MNRJ 41823, Brazil), the female of *Rhopalurus debilis* (Borelli, 1910) from the state of Ceará, Brazil (MIZSUT, now MRSN, Italy), and other specimens of *Physoctonus debilis* (C. L. Koch, 1840) were from Brazil, Pernambuco, Tacaratu (Fazenda Paquiú), 15/VII/2005 (G. Freitas leg.), 2 males (UFPE – MNHN); Maranhão, Caxias, Reserva ecológica, 2/X/2004 (F. Limeira-de-Oliveira), 2 females (MNHN); idem, 5-7/VI/2009 (F. Limeira-de-Oliveira, 1 female (MNHN).

**Museum depository abbreviations are as follows**:

**MNRJ** Museu Nacional, Rio de Janeiro;

**MIZSUT** Museo ed Istituto di Zoologia Sistematica della Università, Torino (now MRSN = Museo Regionale di Scienze Naturali di Torino);

**UFPE** Universidade Federal de Pernambuco;

**MNHN** MMuséum national d’Histoire naturelle, Paris.

## Taxonomic treatment

Esposito et al. (2017) proposed important modifications in the classification of the scorpions associated to the genera *Rhopalurus, Physoctonus*, and *Troglorhopalurus* Lourenço, Cerqueira Baptista & Giupponi, 2004 within a redefined subfamily Rhopalurusinae. In this difficult and sometimes confusing publication, the authors propose a division of the genus *Rhopalurus* creating what I would define as artificial generic groups. Many previously described species are also placed in synonymy, sometimes without the examination of the type material. Worse, some of their conclusions can have a sarcastic tone and push the boundaries of ethics, such as in cases where original type localities are considered as not reliable, naturally based on pure speculation or by stating that other authors published in “obscure journals”. However, these matters will be considered in more details in future publications since the present contribution focuses only on the genus *Physoctonus*.

The genus *Physoctonus* as treated by Esposito et al. (2017) includes the description of a new species, but also contains a number of inaccuracies concerning the diagnoses, ranges of distribution, and ecological aspects, maybe due the lack of sufficient communication among the authors. In this sense, a few points need attention: i) in more than one passage, the revalidation of the genus *Physoctonus* is attributed to Lourenço 2002, when the correct date is 2007; ii) the distribution of *P. debilis* is recorded in the states of Bahia, Ceará, Paraiba, Pernambuco and Piauií (page 89) but in page 93 Bahia is no longer listed. More confusingly, in the map, figure 9B, at least two sites in Maranhão state are both plotted, but no material from Maranhão was examined by the authors. Maybe they refer to the records in Lourenço (2007). The description of the new species *P. striatus* should correspond to *Physoctonus* sp. cited by Yamaguti (2011) in his unpublished thesis. However, Yamaguti (2011) refers the new species to material collected in Oeiras, Piauí, but in the paper by Esposito et al. (2017) the new species is described from Xique-Xique, Bahia, a location of approximately 400 km distance from Oeiras. Moreover, in the abstract of their paper, Esposito et al. (2017) cite Castelo do Piaui as the type locality of *P. striatus*. The material from Oeiras cited by Yamaguti (2011) as new is listed by Esposito et al. (2017) as *P. debilis*. Again, these errors may be the result of a lack of coordination among authors, but are unacceptable, as is this change of the type locality.

In most of their critical remarks of previous papers, Esposito et al. (2017) are very dismissive on the use of colouration patterns by other authors in their diagnoses of new species. Nevertheless, this character is largely used in their diagnosis of *P. striatus*. The use of colour photos in their publication would have been very helpful to illustrate their new species. *Physoctonus striatus* is also defined on the basis of other characters: in particular the position of trichobothrium **db** of pedipalpal chela fixed finger. According to Esposito et al. (2017), **db** is aligned with trichobothrium **et** in *P. striatus* but situated distal to **et** in *P. debilis* - but no illustrations are provided for these characters. In the specimens of *P. debilis* I examined from Pernambuco, trichobothrium **db** is slightly distal or almost aligned with **et**. In the specimens I examined from Maranhão, presumably conspecific with *P. debilis*, trichobothium **db** is clearly basal to **et** whereas in the new species I describe in this work, **db** is only slightly basal to **et** (Fig. 6E–F). Most certainly, the variability of this character should be tested. However, since I did not examine the type material of *P. striatus*, I will presume, a priori, that this is a valid species.

### Family Buthidae C. L. Koch, 1836.

**Genus *Physoctonus* Mello-Leitão, 1934**

#### Physoctonus amazonicus sp. n.

Taxon classificationAnimaliaScorpionesButhidae

http://zoobank.org/1D016803-F350-4481-8621-4B7310E8C445

Figs 4 , 5 , 6

##### Diagnosis.

Medium to small sized scorpion, measuring 28.4 mm in total length for female. General colouration yellow with a brownish inverted triangle covering the anterior margin of carapace; well-marked brownish spots over the lateral edges of carapace and tergites; tergites with a median longitudinal spot which becomes confluent on III to VI; ventral aspect of metasomal segments and appendages intensely spotted. Median ocular tubercle anterior to the centre of carapace; three pairs of lateral eyes. Chelicerae with dentition following the buthid pattern; movable fingers with two small basal teeth, not fused (Vachon 1963). Pedipalps fixed and movable fingers with 7–8 linear rows of granules, and inconspicuous internal and external accessory granules. Sternum between subtriangular and subpentagonal. Pectines small with 13–14 teeth; no stridulatory apparatus; fulcra moderately marked; basal middle lamellae not dilated in female; basal piece strongly marked. Sternites with short linear spiracles. Telson with a globular vesicle; aculeus long and strongly curved, with a vestigial spinoid subaculear tooth. Trichobothrial pattern of type A-α (alpha) – orthobothriotaxic (Vachon 1974, 1975) Tibial spurs absent; pedal spurs reduced.

**Figure 4.**
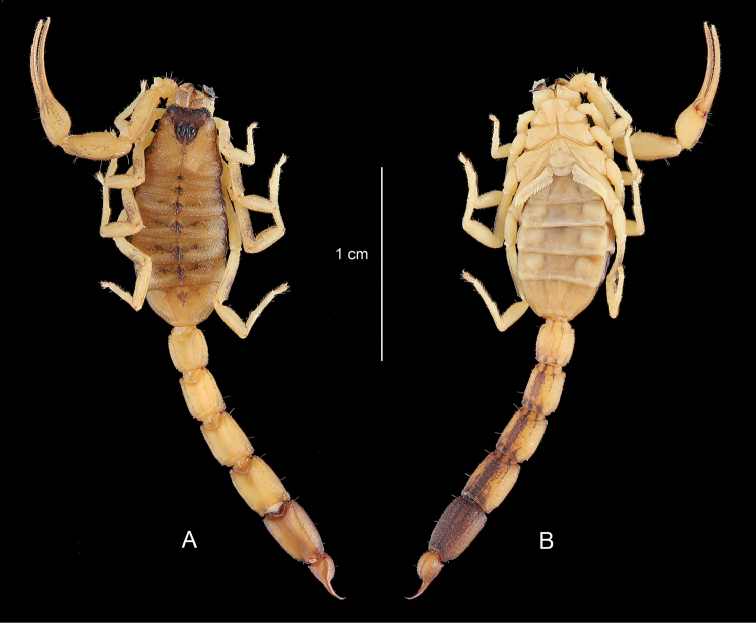
*Physoctonus amazonicus* sp. n., Habitus of female holotype, dorsal and ventral aspects.

**Figure 5.**
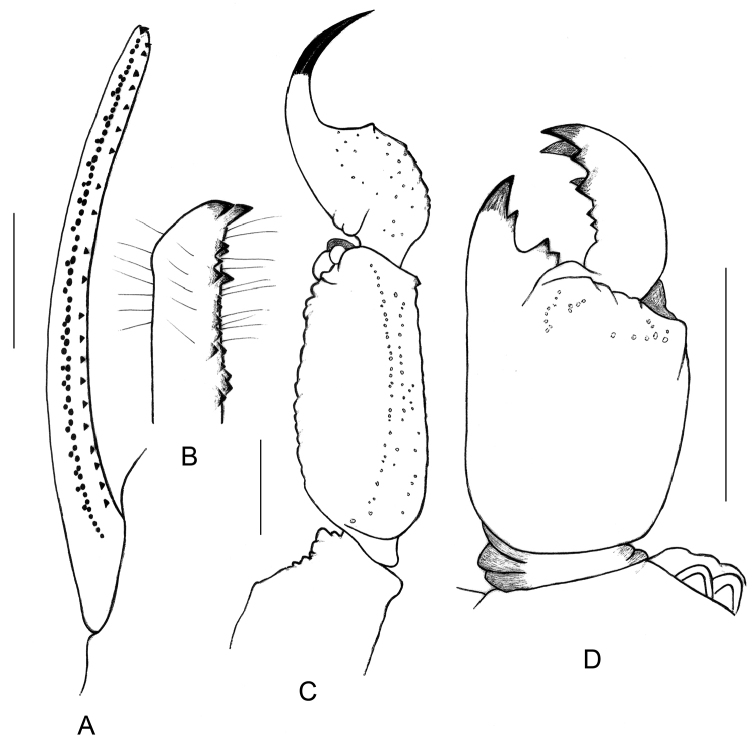
*Physoctonus amazonicus* sp. n., female holotype. **A** Cutting edge of chela movable finger **B** Idem, extremity in detail **C** Metasomal segment V and telson, lateral aspect **D** Chelicera, dorsal aspect. Scale bars: 1 mm.

**Figure 6.**
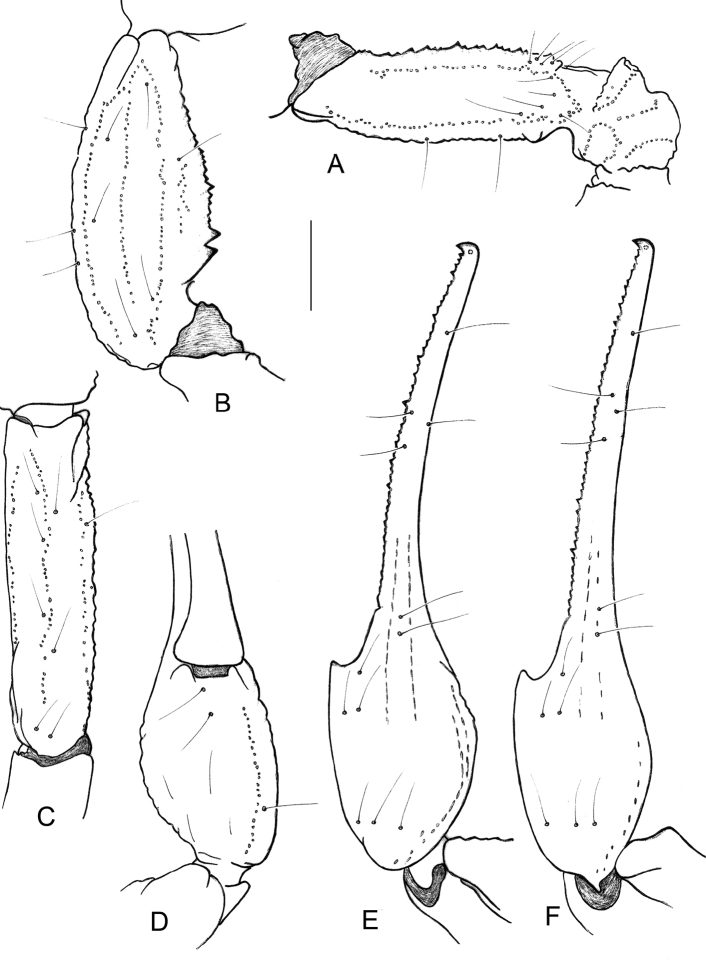
*Physoctonus amazonicus* sp. n., female holotype. Trichobothrial pattern. **A** Femur, dorsal aspect **B–C** Patella, dorsal and external aspects **D–E** Chela, ventral and dorso-external aspects **F** Idem, *Physoctonus debilis*, female from Caxias. Scale bar: 1 mm.

##### Type material.

**Figure 7.**
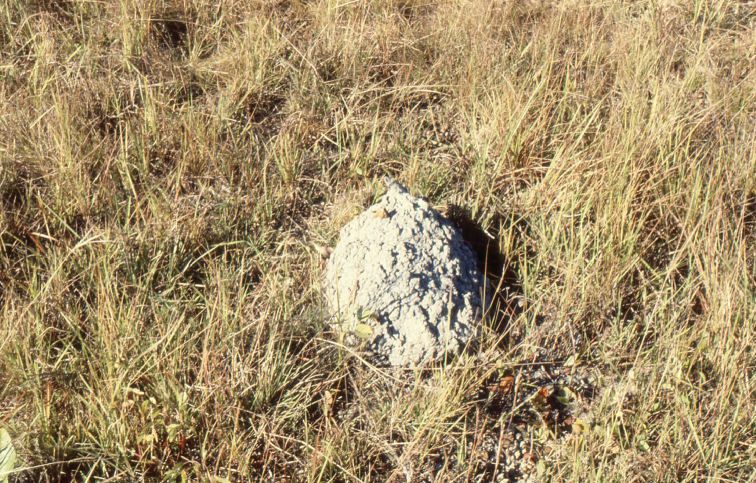
Termite mound of *Armitermes* sp., natural habitat of the new species in the Campos of Pará (photograph by the author, 1979).

Brazil, State of Pará, Campos do Pará, region between rivers Xingu and Araguaya, under termite mound (Fig. 7), XII/1929 (J. Vellard). One female holotype deposited in the collections of the Muséum national d’Histoire naturelle, Paris.

##### Etymology.

The specific name refers to Amazonia, region where the new species was found.

##### Relationships.

**Figure 8.**
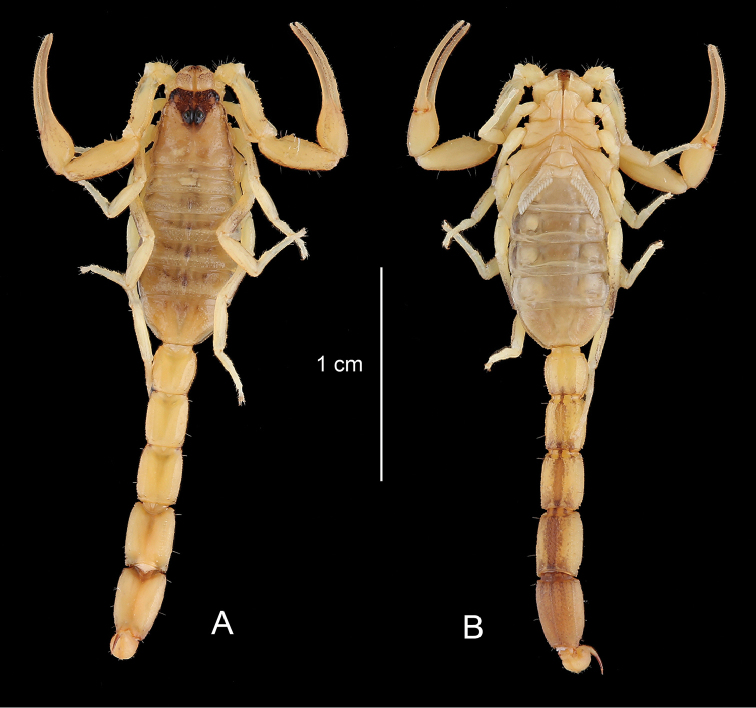
*Physoctonus debilis*. Habitus of female from Caxias, Maranhão, Brazil, dorsal and ventral aspects.

The new species seems to be closer related to *Physoctonus debilis* (C. L. Koch, 1840). Both species can, however, be distinguished by a number of features: (i) a darker general colouration with pigmentation and spots more clearly marked on legs, pedipalps, lateral edges of carapace, tergites, and ventral aspect of metasoma (Figs 4–8), (ii) a more flattened body with a larger mesosoma, (iii) a more strongly marked basal piece, (iv) telson aculeus more strongly curved, (v) trichobothrium **db** of fixed finger only slightly basal to **et**. Moreover, the two species are found in different habitats and are geographically isolated (see ecological and biogeographical comments).

##### Description.

Based on female holotype. Measurements after the description.

Colouration. Basically yellow strongly marked with brownish spots. Prosoma: carapace yellow with a brownish inverted triangle covering the anterior margin of carapace and well-marked brownish spots over the lateral edges; eyes surrounded with black pigment. Mesosoma: tergites yellow with a median longitudinal spot which becomes confluent on III to VI; lateral edges equally spot as for carapace. Metasomal segments I to IV yellow; V reddish-yellow to reddish-brown; segments II to V strongly infuscate ventrally; carinae dark. Vesicle of same colour as segment V; aculeus yellow at the base and reddish on tip. Venter yellow; genital operculum and pectines pale yellow. Chelicerae yellow with a brownish thread; fingers yellow with reddish teeth. Pedipalps yellow with intense but diffused brownish spots; carinae and granulations on the edge of fingers reddish. Legs yellow with intense diffused brownish spots.

Morphology. Carapace strongly granular but with a thin granulation; anterior margin with a median concavity. Anterior median and posterior median carinae moderate to weak. All furrows moderately to weakly deep. Median ocular tubercle distinctly anterior to the centre of the carapace. Eyes separated by approximately one ocular diameter. Three pairs of lateral eyes. Sternum subtriangular to subpentagonal. Mesosoma flattened and enlarged; tergites moderately to strongly granular. Median carina moderate to strong in all tergites. Tergite VII pentacarinate. Venter: genital operculum divided longitudinally, forming two oval plates; basal piece strongly marked. Pectines with 13–14 teeth. Sternites smooth with short linear spiracles; sternite III with some punctations; sternite VII with four carinae and minute granulations. Metasomal segments I to III with ten carinae; IV with eight carinae; V with five carinae; intermediate carinae totally incomplete on segment III. Intercarinal spaces weakly granular on segments I to IV; more clearly marked on V. Telson roughly granular with a long and strongly curved aculeus, slightly shorter than vesicle. Subaculear tooth vestigial. Cheliceral dentition characteristic of the family Buthidae; basal teeth on movable finger reduced but not fused; ventral aspect of both fingers and manus with dense, long setae (Vachon 1963). Pedipalps: femur pentacarinate; patella with seven carinae; chela with nine carinae, weakly marked; internal aspects of femur and patella with several spinoid granules; all faces weakly granular. Fixed and movable fingers with 7–8 linear rows of granules and weakly marked internal and external accessory granules. Trichobothriotaxy: orthobothriotaxy A- (alpha) (Vachon 1974, 1975). Legs: tarsus ventrally with numerous short fine setae; pedal spurs moderate; tibial spurs absent.

Comparative morphometric values (in mm) of an adult female of *Physoctonus debilis* from Caxias Maranhão state and the female holotype of *Physoctonus amazonicus* sp. n. Total length, 28.3/28.4 (including telson). Carapace: length, 3.7/3.6; anterior width, 2.3/2.4; posterior width, 3.9/4.4. Mesosoma length, 8.6/8.2. Metasomal segments. I: length, 2.0/2.1; width, 2.2/2.1; II: length, 2.3/2.5; width, 1.9/2.0; III: length, 2.6/2.7; width, 2.1/2.0; IV: length, 3.0/3.1; width, 2.2/2.2; V: length, 3.1/3.4; width, 2.2/2.2; depth, 1.6/1.4. Telson: length, 3.0/2.8; width, 1.2/1.3; depth, 1.2/1.2. Pedipalp: femur length, 3.5/3.4, width, 1.2/1.1; patella length, 3.9/3.7, width, 1.6/1.5; chela length, 6.8/6.7, width, 1.3/1.5, depth, 1.0/1.2; movable finger length, 4.6/4.5.

### Biogeographical and ecological considerations

The genus *Physoctonus* is a typically Neotropical element distributed only in the north range of South America (Fig. 3). The only two species included in this genus until now, *P. debilis* and *P. striatus* seem to be typical elements of the caatinga formations of the north-east of Brazil (Esposito et al. 2017), but the population from Maranhão is located in a transitional area, between cerrados and matas (Lourenço in prep.). Apparently the two previously known *Physoctonus* species are only found under stones and bark of the xerophytic vegetation, and under logs in the sites of Maranhão. Contrarily, the new species described at present was found under a termite mound of *Armitermes* sp., a typical microhabitat of cerrados and campos from Central Brazil and southern Amazonia (Lourenço 2000).

With the discovery of a third species of *Physoctonus* in the Campos of southern Amazonia (Fig. 2), it seems possible to establish a parallel between this pattern of distribution and the one presented by the species of the genus *Rhopalurus* (sensu Lourenço, 2007). For details on the ecology of these Amazonian savannahs refer to Lourenço (2000). In both cases, these two genera constitute suitable examples of groups presenting discontinuous distributions over open vegetation formations. These examples have an important relationship with species endemic to present islands of savannah in Amazonian and Guayanian enclaves (Mori 1991). The endemic populations isolated inside savannah islands provide good evidence in support to the hypothesis of past connections between the savannahs and Caatingas of Central and northeastern Brazil and the savannah enclaves in Amazon and Guayana regions. During past palaeoclimatic vicissitudes associated with major dry periods, forest cover was reduced; open vegetation formations probably coalesced during these climatic events which took place at the end of Cenozoic and during Pleistocene times (Ab’Saber 1977; Van der Hammen 1983).

Scorpion patterns of distributions represent good examples to support this hypothesis. The species of *Physoctonus* (as those of the genus *Rhopalurus*) most certainly exhibited a continuous distribution during Pleistocene dry periods and the present disrupted distribution is a possible consequence of the reestablishment of rainforest over the regions which previously served as corridors (Lourenço 1986, 2001b, 2008). In the case of *Rhopalurus*, at least one species *R. amazonicus* Lourenço, 1986 is endemic to a savannah enclave in the region of Alter do Chão (State of Pará, Brazil) in total isolation within oriental Amazon forest (Murça-Pires and Prance 1985). This example offers additional support to the theory of disrupted distributions. The present description of a new species of *Physoctonus* from the Campos of southern Amazonia seems to conform to this biogeographic model.

This rather recent geographic isolation probably led to a minor process of speciation and differentiation, and as consequence the Amazonian population now found in an isolate fragment of savannah shows little morphological differences. In face of the observed patterns of distribution and differentiation it becomes difficult to be certain about the true taxonomic status of some isolated populations. Consequently one question can be addressed: are these populations true species, subspecies, or only local morphs belonging to large polymorphic populations?

The consequences on the process of speciation during the subsequent wet/dry/wet periods is difficult to measure, but probably they were rather weak on groups such as scorpions with long term reproduction process and a low number of generations when compared to other zoological groups such as insects (Prance 1982; Lourenço 2001b). It is therefore difficult to assign a precise status to the two allopatric populations. Are we in face of species, subspecies or only morphs of a large polymorphic species? If their specific condition could be demonstrated in association with a clear allopatric distribution of the populations, then the condition of super-species sensu Mayr (1931) may be applied to each of the two lineages. The species within each lineage would be represented by allopatric, parapatric, or weakly sympatric groups, really or potentially intersterile in nature (Bernardi 1980). Each of these species could then be defined as a prospecies in the sense of Birula (1910).

## Supplementary Material

XML Treatment for Physoctonus amazonicus
